# Intraoperative Myelography in Cervical Multilevel Stenosis Using 3D Rotational Fluoroscopy: Assessment of Feasibility and Image Quality

**DOI:** 10.1155/2015/498936

**Published:** 2015-08-02

**Authors:** Thomas Westermaier, Stefan Koehler, Thomas Linsenmann, Michael Kiderlen, Paul Pakos, Ralf-Ingo Ernestus

**Affiliations:** Department of Neurosurgery, University Hospital Wuerzburg, Josef-Schneider-Strasse 11, 97080 Wuerzburg, Germany

## Abstract

*Background*. Intraoperative myelography has been reported for decompression control in multilevel lumbar disease. Cervical myelography is technically more challenging. Modern 3D fluoroscopy may provide a new opportunity supplying multiplanar images. This study was performed to determine the feasibility and image quality of intraoperative cervical myelography using a 3D fluoroscope. *Methods*. The series included 9 patients with multilevel cervical stenosis. After decompression, 10 mL of water-soluble contrast agent was administered via a lumbar drainage and the operating table was tilted. Thereafter, a 3D fluoroscopy scan (O-Arm) was performed and visually evaluated. *Findings*. The quality of multiplanar images was sufficient to supply information about the presence of residual stenosis. After instrumentation, metal artifacts lowered image quality. In 3 cases, decompression was continued because myelography depicted residual stenosis. In one case, anterior corpectomy was not completed because myelography showed sufficient decompression after 2-level discectomy. *Interpretation*. Intraoperative myelography using 3D rotational fluoroscopy is useful for the control of surgical decompression in multilevel spinal stenosis providing images comparable to postmyelographic CT. The long duration of contrast delivery into the cervical spine may be solved by preoperative contrast administration. The method is susceptible to metal artifacts and, therefore, should be applied before metal implants are placed.

## 1. Introduction

Patients with multilevel spinal stenosis present a diagnostic and operative challenge. If surgery is indicated, the necessary decompression has to be weighed up against the loss of stability [1]. In these patients, intraoperative control of appropriate surgical decompression is of interest and intraoperative imaging may be helpful for this purpose. In lumbar spinal procedures, decompression control has previously been assessed by intraoperative myelography [2]. Cervical myelography is technically more challenging and there have been no reports about its intraoperative use. In the last years, intraoperative 3D imaging has been introduced in surgical operating theaters. It has mainly been used for osteosynthesis control [3, 4]. However, its ability to acquire good quality multiplanar images may also be useful for decompression control when combined with intrathecal contrast agent. This study investigated the feasibility, image quality, and diagnostic value of intraoperative 3D fluoroscopic myelography for decompression control in patients with multilevel stenosis of the cervical spine.

## 2. Materials and Methods

This retrospective analysis was in accordance with the guidelines of the institutional ethics committee. Before surgery, the option of intraoperative decompression control had been discussed with the patients. They were distinctly informed about the potential risks of lumbar puncture and placement of a lumbar drainage, including the risk of nerve root injury, the risks of administration of an iodine contrast agent, and radiation exposure, and a possible prolongation of the surgical procedure caused by intraoperative myelography. All patients gave informed consent.

### 2.1. Inclusion Criteria

Patients met the inclusion criteria if they were over 18 years and had stenosis of the cervical or upper thoracic vertebral canal with the indication for surgical treatment. Patients were excluded if they had a history of allergy** or intolerance** against iodine contrast agent or renal insufficiency or if the serum creatinine value was above 100 *μ*mol/L (1.2 mg/100 mL).

### 2.2. Patient Positioning and Image Acquisition

Prior to surgery, a lumbar drainage was implanted under general anesthesia. If posterior decompression was performed, the patient's head was fixed in a radiolucent carbon Mayfield clamp and positioned on a radiolucent operating table in a prone position. In case of anterior decompression, the patients were positioned on the radiolucent operating table in a supine position.

**Figure 1:**
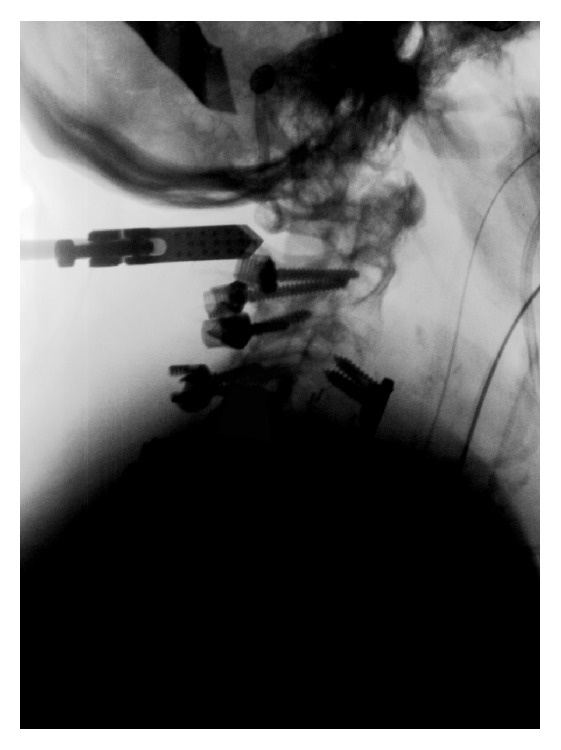
Lateral fluoroscopic view depicting the contrast delivery into the cervical spinal canal.

After decompression, 10 mL of iodine contrast agent (Isovist 240) was administered via the lumbar drainage. Then, the operating table was tilted head down in order to allow the contrast agent to flow into the cervical canal until serial lateral or anteroposterior fluoroscopy using the 3D fluoroscope (O-Arm, Medtronic) showed the intrathecal contrast flow (Figure 1). When the cervical canal was filled with contrast agent, a 3D rotational fluoroscopy scan was performed. Thereafter, the table was readjusted and the operation continued. For 3D image acquisition, the following O-Arm settings were used: “High Definition Mode”; gantry tilt 0 degrees; gantry rotation 360 degrees; image acquisition time 24 seconds; reconstruction time 24 seconds; standard O-Arm collimator thickness without additional collimation; digital flat panel detector 40 × 30 cm, camera resolution 2000 × 1500 (3 megapixels); pixel pitch 0.194 mm; reconstruction matrix 512 × 512 × 192).

### 2.3. Evaluation of Images

The images were visually assessed during the operation by the surgical team. Sufficient decompression was usually assumed if a continuous contrast-enhanced layer of cerebrospinal fluid (CSF) surrounded the spinal cord. In case of residual stenosis decompression was continued. For analysis of image quality, images were transferred to an Apple PowerMac workstation using OsiriX freeware and reassessed by two of the coauthors (Thomas Linsenmann, Stefan Koehler) using the following four-grade scale: −: spinal canal and cord not visible/assessable; (+): spinal canal and contrast agent poorly visible/assessable; +: spinal canal and contrast filling clearly visible/assessable. Postoperative CT and/or MRI was assessed by the same persons for residual stenosis. The clinical course after surgery was assessed using the European Myelopathy Score (EMS).

## 3. Results

### 3.1. Patient Characteristics

Ten surgical procedures in the cervical spine were performed in *n* = 9 patients using intraoperative myelography. All patients had cervical, cervicothoracic, or upper thoracic spinal stenosis. Patient characteristics are depicted in Table 1. In six patients, a posterior approach was performed, and in two patients an anterior approach was performed. One patient underwent both anterior and posterior surgery.

### 3.2. Side Effects

The implantation of lumbar drainage and the administration of intrathecal contrast agent were not followed by any unwanted side effects. In particular, no anaphylactic reaction or seizures occurred. No patient developed meningitis or other infectious complications and no symptoms due to a possible loss of CSF were recorded after surgery. Furthermore, there were no complications secondary to the implantation of a lumbar drainage.

### 3.3. Workflow and “Time-to-Arrival”

The 3D fluoroscope was positioned during preoperative preparation (shaving, disinfection). The contrast agent was administered after decompression was apparently completed. The time until the contrast agent arrived in the cervical spinal canal was 15 ± 12 min in posterior approaches and 25 ± 8 min in anterior approaches.

### 3.4. Image Quality

**Figure 2:**
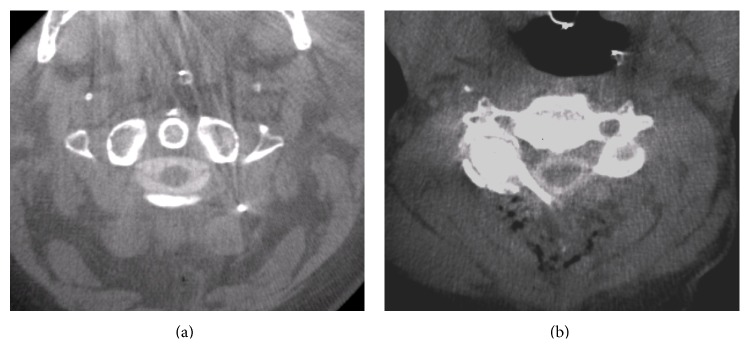
Without metal artifacts, good quality images can be obtained comparable to CT myelography. Transverse views of case 4 (a) and case 8 (b).

**Figure 3:**
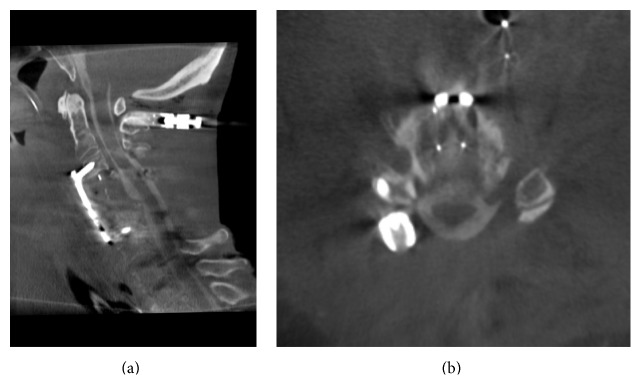
Sagittal (a) and transverse (b) view of intraoperative myelography after anterior and posterior decompression. The images were acquired after posterior instrumentation and depict the susceptibility to metal artifacts.

**Figure 4:**
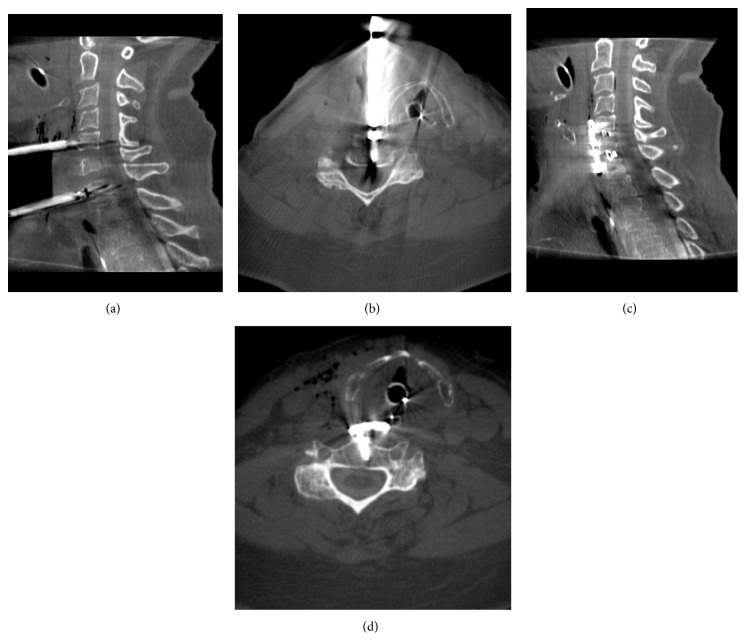
Intraoperative myelogram obtained for the purpose of decompression control. Vertebral bodies were distracted with Caspar-pins ((a) and (b)) which caused a considerable artifact making the assessment of sufficient decompression difficult. After removal of the Caspar-pins and implantation of intervertebral cages at the disc levels C5/6 and C6/7 and instrumentation with a plate screw osteosynthesis ((c) and (d)), the assessment of the extent of decompression is better with reduced metal artifacts.

Image quality was excellent if 3D scans were acquired prior to metal implantation (Figures 2(a) and 2(b)). Contrast enhancement was better in posterior approaches than in anterior approaches. Metal artifacts considerably reduced image quality, especially in posterior procedures (Figures 3(a) and 3(b)). Caspar-pins used for vertebral distraction in anterior approaches also caused strong metal artifacts and reduced image quality (Figures 4(a)–4(d)). The results of the assessment of image quality and the operative consequences drawn from intraoperative myelography are depicted in Table 1. In four of the 10 procedures, the operative strategy was changed after the evaluation of intraoperative myelograms.

### 3.5. Clinical and Radiological Follow-Up

Of all patients, postoperative radiological follow-up examinations were available. Seven patients were followed up by MRI, and two patients by CT. In all cases, an excellent decompression without residual stenosis was found. Seven patients showed neurological improvement after surgery, and one patient remained unchanged. One patient died from peritonitis 6 weeks after surgery (Table 1).

## 4. Discussion

To the best of our knowledge, this is the first report about the use of intraoperative contrast-enhanced 3D fluoroscopy for cervical myelography. The results of this series show that intraoperative 3D fluoroscopic myelography can generate images of good quality comparable to CT myelography (CTM). Similar to computed tomography (CT), however, the technology is rather susceptible to metal artifacts. The interval between administration of the contrast agent via lumbar drainage and its arrival interrupts surgery and is a further drawback to be eliminated.

The general value of myelography as a diagnostic method for the evaluation of spinal diseases has been much disputed in the recent years. Since magnetic resonance imaging (MRI) has become the standard examination for the diagnosis of spinal diseases, the role of myelography combined with CTM, a standard procedure in the pre-MRI era, has decreased. Its use for diagnostic purposes varies significantly depending on surgeon and department. Classical indications are patients carrying pacemakers and multilevel spinal stenosis with incongruent clinical and radiological findings.

For intraoperative use, the situation is different. Intraoperative MRI is poorly practicable for the control of operative results in spine surgery. Fluoroscopy is the gold standard for this purpose, particularly for the control of implant positions. Intraoperative myelography using biplanar fluoroscopy, in contrast, has long been used for particular cases of lumbar spinal diseases [5, 6]. Intraoperative 3D fluoroscopy is novel technique which provides good quality images and better comparability with preoperative tomographic examinations. Mauer and coworkers detected residual stenosis in 2 of 10 patients who underwent unilateral laminotomy for the treatment of lumbar degenerative disease using intraoperative 3D fluoroscopy for decompression control [7]. The authors further concluded that intraoperative myelography, which had been performed in 5 of 10 patients, gave no additional information. However, in at least one patient, residual stenosis had been detected using contrast-enhanced 3D fluoroscopy. Unfortunately, there is no information whether the second patient with residual stenosis had also received contrast agent. Thus, the benefit of additional myelography to 3D fluoroscopy cannot be extracted from this series. Myelography provides direct information about the compression of neural structures in the spinal canal. Spinal stenosis is caused not only by bony structures but also by hypertrophic ligaments and facet joint capsules and prolapsed disk material which are unlikely to be detected by nonenhanced 3D fluoroscopy [8–11]. Therefore, it is likely that myelography may change the operative strategy in a number of patients as compared to nonenhanced 3D fluoroscopy. Patel et al. reported intraoperative myelography for the control of adequate decompression in a series of 10 patients. Three patients required further decompression. The authors reported a delay of surgery between 10 and 20 minutes and considered the technique to be safe and efficient [12].

Sembrano and coworkers recently reported intraoperative 3D fluoroscopy in 100 thoracic and lumbar spine procedures [13]. In a part of their patients collective decompression control was the target of intraoperative imaging. 19 of these patients received intrathecal contrast agent for this purpose. In 6 patients, decompression was extended after an initial 3D rotational scan, which underlines the usefulness of intraoperative imaging in complex cases. Altogether, the authors reported a 20% rate of change of the surgical procedure after intraoperative image control. The numbers of patients in the abovementioned reports are small. However, the rate of residual stenosis seems to be between 20 and 30% in the lumbar spine if assessed by intraoperative myelography using 3D fluoroscopy. On first sight, these numbers seem to be rather high. Apparent residual stenosis, as depicted by 3D fluoroscopy or myelography, might not absolutely correlate with postoperative complaints. Evaluating the clinical benefit of decompression control by intraoperative imaging in terms of postoperative pain or neurological recovery requires a prospective study design and a large number of patients. However, incomplete decompression has been reported to be one reason for poor results after spinal decompression [14]. The present series is a technical report of 10 operations in patients with cervical stenosis. In 4 patients, the operative strategy was changed after intraoperative myelography. Structures in the cervical spine are smaller and a pronounced cervical lordosis bears the risk of under- or overestimation of the width of the spinal canal on the upper or lower limit of decompression and, therefore, intraoperative imaging may detect an even higher rate of residual stenosis in cervical stenosis, particularly in limited decompression surgery.

### 4.1. Technical Pitfalls and Limitations

Cervical myelography differs from the lumbar myelography in that the distance the contrast agent needs to be delivered is longer and includes the thoracic kyphosis. For that reason, diagnostic cervical myelography is usually performed in a lateral position. Contrast delivery to the cervical canal is challenging in the intraoperative setting when patients are in a prone or supine position. The operating table must be sharply tilted and the patient firmly fixed to maintain his position. During these 10 intraoperative myelographies, we noticed a learning effect, which especially concerned the sufficient tilting of the operating table and the implementation of myelography into the operative workflow and which reduced the prolongation of surgery. This is in accordance with the findings of Patel et al. who also reported a steep learning curve using intraoperative lumbar myelography in 10 patients with lumbar stenosis [12]. After a more frequent use, a further marked acceleration of the procedure can be expected. As the patient characteristics illustrate, all patients of this series were older than 60 years. Although we did not perform a systematic correlation of contrast delivery time and changes in the lumbar spine, older patients have an increasing natural risk to develop degenerative lumbar stenosis. This may have additionally prolonged the contrast delivery into the cervical region, a phenomenon which is well known from diagnostic myelography.

In anterior approaches, it may be particularly difficult to surmount the thoracic kyphosis and shift the contrast agent into the cervical canal. Therefore, its delivery into the region of interest takes markedly longer in supine than in prone position and the amount of contrast agent arriving in the cervical canal is lower. This is likely to be the reason for a poorer contrast enhancement in anterior approaches. Earlier administration of the contrast agent via the lumbar drainage, for example, at the beginning of decompression or even prior to surgery in a lateral position, will possibly solve this problem.

This series demonstrates that in the presence of osteosynthesis material image quality decreases due to metal artifacts. After anteroposterior instrumentation, the spinal canal may be not assessable at all. Therefore, decompression control before instrumentation must be recommended when using 3D fluoroscopy. Alternatively, image subtraction of native and contrast-enhanced images may solve this problem. Particular attention must be given to Caspar-pins used for vertebral distraction in anterior approaches. The way they are usually placed causes a line-shaped artifact which covers the region that is meant to be assessed for intraoperative decompression control.

## 5. Conclusions

The number of changes of the operative strategy secondary to intraoperative myelography in our series underlines that this technique is useful for the intraoperative assessment of residual stenosis. Myelography not only directly visualizes the compression of neural structures but also depicts the intraspinal anatomic conditions after patient positioning which is particularly valuable in patients planned to receive spondylodesis. Metal artifacts seriously deteriorate image quality. Thus, myelography prior to the placement of osteosynthesis material must be recommended. Similarly, the removal of other metal devices like retractors or Caspar-pins should be advised. The rather long delay of surgery is mainly due to the interval between contrast administration and its arrival in the cervical canal. This patient collective predominantly consists of older patients who are likely to have concomitant lumbar stenosis. The presence of lumbar stenosis may be one reason for the delayed delivery of the contrast agent into the cervical spinal canal. The lack of experience using a novel technique is another reason and is likely to improve after repeated use and shorten the operative delay. Preoperative contrast administration in a lateral position, similar to diagnostic myelography, may further decrease the delay of surgery. However, this has to be weighed up against the wash-out time of the intrathecal contrast agent which might be more rapid than operative decompression. These issues will be investigated in a further prospective study.

## Figures and Tables

**Table 1:** Characteristics and surgical approach in *n* = 9 patients undergoing intraoperative myelography. One of the patients had upper thoracic stenosis due to osteomyelitic vertebral body fracture. Myelography was performed to evaluate if the extent of decompression was sufficient. Image quality largely depends on metal artifacts and if the thoracic kyphosis can be overcome (OPLL = ossification of the posterior longitudinal ligament, VA = vertebral artery, CSM = cervical spondylotic myelopathy, f = female, m = male, CT = computed tomography, MRI = magnetic resonance imaging, and EMS = European Myelopathy Score).

Patient number	Name, sex, age	Pathology	Approach	Procedure	Image quality	Change of operative strategy	Residual stenosis in postoperative imaging	Clinical result (EMS before/after surgery
1	D.N., m, 76 y	OPLL (after corpectomy C4–6)	Posterior	Spondylodesis C3–C7	+	Additional laminectomy C4	No (MRI)	12/14

2	K.S., m, 70 y	OPLL	Posterior	Laminectomy C1–C3 Spondylodesis C1–C3	(+)	—	No (MRI)	16/18

3	G.P., f, 69 y	Osteomyelitic fracture T3 and T4	Posterior	Costotransversectomy and vertebrectomy T3 and T4 ventrodorsal spondylodesis	− (metal artifacts)	—	No (MRI)	6/died 6 weeks after surgery due to peritonitis

4	E.M., f, 76 y	CSM, increasing dislocation of C2 fracture 3 months earlier	Posterior	Laminectomy C3–C5 Spondylodesis C2–C6	(+)	—	No (MRI)	17/18

5	W.S., f, 68 y	CSM, aggravated after laminectomy C5	Anterior	Corpectomy C4/5	−	—	No (MRI)	7/12

5	W.S., f, 68 y	CSM, aggravated after laminectomy C5 VA injury during corpectomy	Posterior	Laminectomy C4–C7 Spondylodesis C2–T2	+	Extension of posterior decompression	No (MRI)	7/12

6	E.N., m, 70 y	CSM, discectomy C4/5 1 year earlier	Anterior	Corpectomy C5	+	—	No (MRI)	15/16

7	W.S., m, 62 y	Dislocated C1/2 fracture	Posterior	Spondylodesis C1–3	+	—	No (CT)	17/18

8	R.M., m, 74 y	CSM	Posterior	Laminectomy C3 and C4 Spondylodesis C2–C6	+	Extension of decompression	No (MRI)	13/13

9	M.K., f, 73 y	CSM	Anterior	Discectomy C4/5 and 5/6	+	2-level discectomy instead of corpectomy	No (CT)	11/13
